# Radiosensitization Effect of PARP Inhibitor Talazoparib Involves Decreasing Mitochondrial Membrane Potential and Induction of Cellular Senescence

**DOI:** 10.3390/cimb47110908

**Published:** 2025-11-01

**Authors:** Barkha Saraswat, Ankitha Vadi Velu, Zhongming Gao, Zongxiang Zhang, Haoyang Zhu, Ying Tong, Mitsuko Masutani

**Affiliations:** Department of Molecular and Genomic Biomedicine, Center for Bioinformatics and Molecular Medicine, Nagasaki University Graduate School of Biomedical Sciences, 1-12-4, Sakamoto, Nagasaki 852-8523, Japan; bb55322032@ms.nagasaki-u.ac.jp (B.S.); bb55322801@ms.nagasaki-u.ac.jp (A.V.V.); bb55323014@ms.nagasaki-u.ac.jp (Z.G.); bb55323025@ms.nagasaki-u.ac.jp (Z.Z.); bb55a24010@ms.nagasaki-u.ac.jp (H.Z.); y-tong@nagasaki-u.ac.jp (Y.T.)

**Keywords:** γ-irradiation, radiosensitization, PARPis, talazoparib, senescence

## Abstract

Poly (ADP-ribose) polymerase (PARP) inhibitors (PARPis) with radiation therapy can enhance the sensitivity of cancer cells by inhibiting DNA repair pathways. To determine the most suitable PARP inhibitor for radiosensitization in cancer cells, we compared various types of clinically used PARPis in lung cancer A549 cells. We found that most PARP inhibitors showed radiosensitization effects on A549 cells. ER10 values for talazoparib, olaparib rucaparib, ABT888 and niraparib were 1.5, 1.8, 2.8, 1.4, and 1.4, respectively. Talazoparib showed a radiosensitization effect at its lowest concentration. Talazoparib is a potent PARP inhibitor and has been used in clinical settings for several types of cancer as an anti-cancer agent. We thus focused on how talazoparib causes radiosensitization in lung cancer A549 cells. As a result of the combination of talazoparib and γ-irradiation, we observed an increased level of cellular senescence accompanied by a decrease in mitochondrial membrane potential. When the *p21* gene was knocked down, both the decrease in mitochondrial membrane potential and senescence level were attenuated, suggesting that p21 is involved in senescence induction after γ-irradiation combined with talazoparib treatment. Taken together, we showed that PARP inhibitor talazoparib treatment in combination with γ-irradiation causes cellular senescence in lung cancer cells, involving p21 function.

## 1. Introduction

Radiotherapy is a central treatment for many cancers, including lung adenocarcinoma, but its success is often limited by the ability of tumor cells to repair DNA damage and continue proliferating. Enhancing the effects of radiation through radiosensitization has therefore become an important strategy in improving therapeutic response [1]. Through genome-wide screening, we previously confirmed that poly (ADP-ribose) polymerase 1 (PARP1) is a candidate target for radiosensitization [2]. PARP inhibitors impair DNA repair and magnify radiation-induced cytotoxicity in various types of cancer cells [3,4]. Clinical and preclinical studies with PARP inhibitor olaparib [5,6] have demonstrated that this inhibitor sensitizes tumors to radiation, with evidence of benefits in lung cancer models as well. Given the high DNA repair capacity of lung adenocarcinoma cells, blocking PARP activity may provide a strong rationale for improving radiotherapy outcomes in this tumor type. Beyond DNA repair inhibition, PARP inhibitors are reported to affect diverse physiological processes as well.

For example, other than DNA repair inhibition, PARP inhibitors are reported to change gene expressions [7] and to induce senescence, a state of permanent growth arrest. While senescence can initially suppress tumor progression, in lung adenocarcinoma it may also allow tumor cells to persist in a dormant state and later escape, contributing to recurrence [8]. Understanding the balance between beneficial and harmful aspects of therapy-induced senescence is therefore particularly relevant in this context. Various radiosensitization strategies involve senescence induction [9,10,11,12,13,14] or alternatively, attenuate senescence [15]. Senescence induction is triggered by p53-dependent upregulation of cell cycle regulator p21 or through other cascades. In addition, the mitochondrial membrane potential plays an important role in linking DNA damage to metabolic collapse. Loss of mitochondrial integrity after PARP inhibition and radiation may increase radiosensitivity in lung adenocarcinoma cells [16]. The cell cycle regulator p21 can be a critical factor in enforcing senescence and mediating cell fate after DNA damage; its regulation in this tumor type may determine whether cells undergo stable arrest or recover proliferative potential [17].

Although PARP inhibitors like olaparib and talazoparib enhance radiation responses, studies suggest that lung adenocarcinoma cells may recover proliferative ability, raising the risk of recurrence [18]. This study therefore investigates how PARP inhibitor talazoparib, in combination with radiation, influences senescence and mitochondrial membrane potential through p21 in lung adenocarcinoma cells, aiming to identify strategies that improve therapeutic durability and minimize tumor relapses.

## 2. Materials and Methods

### 2.1. Cell Culture

The human lung cancer A549 cell line was obtained from the American Type Culture Collection. RPMI1640 medium (Gibco, Waltham, MA, USA) containing 10% fetal bovine serum (HyClone, Logan, UT, USA) and 1% penicillin-streptomycin (Gibco) was used as a culture medium.

### 2.2. Chemicals

Talazoparib, olaparib, BSI201, ABT-888 and niraparib were purchased from Selleck Biotech. Rucaparib was obtained from Adoq Bioscience (Irvine, CA, USA).

### 2.3. γ-Irradiation

Exponentially growing cells were irradiated by and ^137^Cs γ-irradiator at a dose rate of 1.0 Gy/min at Nagasaki University (PS-3100SE, Pony Industry, Tokyo, Japan).

### 2.4. Clonogenic Survival Assay

A549 cells were inoculated in 6-well plates, and the next day cells were treated with PARP inhibitors, and after 2 h of incubation at 37 °C, cells were irradiated with ^137^Cs. Nine days after irradiation, colonies were fixed with 10% formalin (Wako, Tokyo, Japan) and stained with crystal violet followed by colony counting. Colonies containing more than 50 cells were counted.

### 2.5. RNAi Experiments

Silencer Select Validated siRNA targeting *p21* (Integrated DNA Technologies, Coralville, IA, USA) was transfected by lipofectamine ^®^RNAi MAX according to the manufacturer’s protocol. Briefly, 1 × 10^5^ cells were seeded to a well of a 12-well culture plate (Thermo Scientific, Waltham, MA, USA) 24 hrs. before transfection. Then, cells were treated with transfection regent lipofectamine ^®^RNAi MAX reagent and siRNA in Gibco Opti-MEM^®^ using reduced serum medium (Life Technologies, Carlsbad, CA, USA).

### 2.6. Cell Senescence Assay

The SA-β-Galactosidase activity was assessed using X-gal as a substrate at 37 °C. It was incubated overnight, followed by PBS (−) washing and observation under a fluorescence microscope. A SPiDER-β-gal assay (Dojindo, Tokyo, Japan) was performed according to the instructions from the manufacturers. Flow cytometry was performed with the FACS Versa system (Becton-Dickinson, Franklin Lakes, NJ, USA).

### 2.7. Level of Mitochondria Membrane Potential Measurement

The level of mitochondrial membrane potential was measured using the fluorescent dye JC-1 (5,5, 6,6’-tetrachloro-1,1’, 3,3’-tetraethyl benzimidalyl carbocyanine iodide), purchased from Dojindo. The ratio of red (585 nm) to green (530 nm) fluorescence represents the changes in mitochondrial membrane potential. A549 cells were stained with 2 μM JC-1 for 30 min at 37 °C after being treated with talazoparib for 3 days. The cells were then trypsinized, JC-1 fluorescence was detected using BD FACS Verse (Beckton and Dickinson) flow cytometry, and data were analyzed using Flow Jo software (v10.10).

### 2.8. Statistical Analysis Methods

Data were expressed as mean ± S.E. values. In this study, data were analyzed using Tukey’s test with JMP Pro 17 software. Statistical significance was indicated when the value was less than 0.05.

## 3. Results

### 3.1. Radiosensitization Effects of Various PARPi on A549 Cells

**Figure 1:**
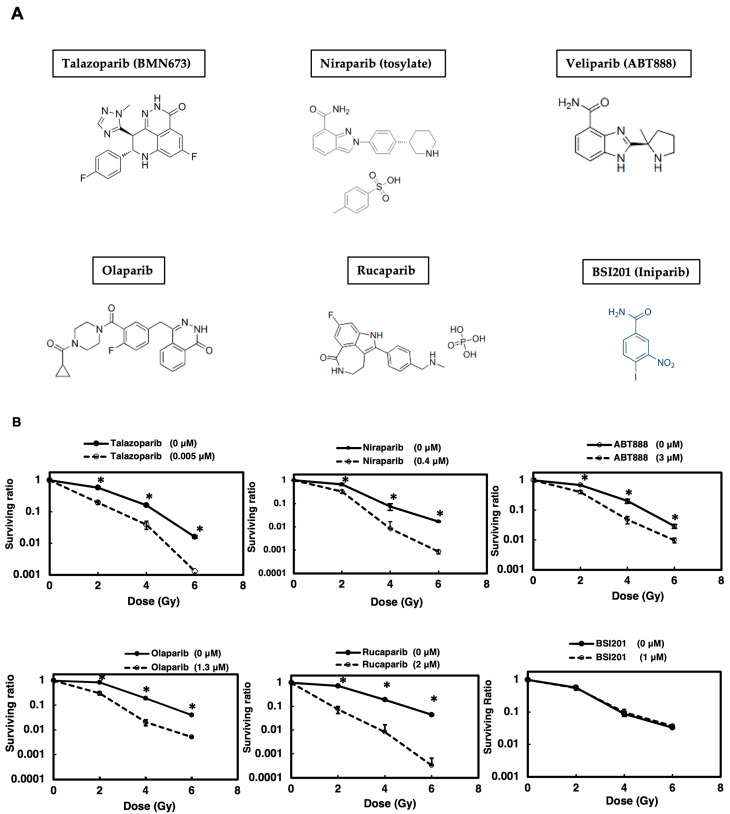
PARP inhibitors (**A**) except for BSI201 showed radiosensitization effects on A549 cells. A colony formation assay was performed to assess the radiosensitizing effect of various PARP inhibitors on A549 cells (**B**). Cells were treated with different PARP inhibitors and exposed to γ-irradiation. Cell survival ratios were determined. Data represent mean ± SE of three independent experiments. * *p* < 0.05.

To investigate the impact of various PARP inhibitors in radiosensitization therapy, we focused on lung cancer, which is frequently treated with radiation therapy. We used A549 lung adenocarcinoma cells as a model, which retain a p53-dependent signal transduction pathway. As illustrated in Figure 1A,B, when a clonogenic survival assay was carried out after γ-irradiation (Table 1) following a 2 h pretreatment with PARP inhibitors, the various clinically used PARP inhibitors all exhibited a radiosensitization effect, except for BSI201. This was expected, as BSI201 is a weak PARP inhibitor and lacks the ability to directly influence PARP activity. Among the inhibitors used, talazoparib demonstrated the greatest efficacy at the lowest concentrations, in the range of 5 nM. Notably, the results indicate that rucaparib achieved a higher enhancement ratio at 10% survival (ER10), whereas niraparib exhibited a comparatively lower ER10 (Table 2). Moving forward, our focus centered on studying the mechanism of action of talazoparib in more detail.

### 3.2. Cell Cycle Analysis After Talazoparib and γ-Irradiation at 24 h and 72 h of Treatment

**Figure 2:**
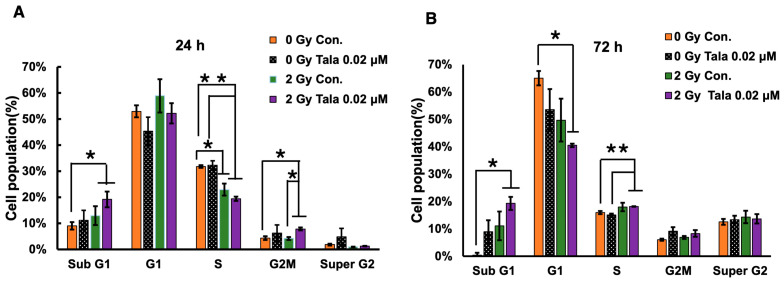
Cell cycle analysis 24 and 72 h after combined γ-irradiation and talazoparib treatment. A cell cycle analysis was conducted following the combined treatment of A549 cells with talazoparib at a concentration of 0.02 µM and gamma irradiation, with a sample size of *n* = 3 (biological replicates), at 24 (**A**) and 72 h (**B**) post-treatment. The cell cycle distribution percentage is shown as the mean ± SE. Statistical significance is indicated by * *p* < 0.05, ** *p* < 0.01. Supplementary Figure S1 shows cell cycle profiles.

The colony formation assay results suggest that low concentrations of talazoparib with combined treatments of irradiation were more effective at reducing the survival of A549 lung cancer cells. To analyze the effect of talazoparib on cell cycle distribution and apoptosis, a cell cycle analysis was carried out as shown in Figure 2A,B. It showed that the combination of γ-irradiation and talazoparib in A549 caused the S phase and G1 phase to decrease, whereas a slight increase in the apoptotic sub-G1 phase can be seen 24 h and 72 h after treatment. This apoptosis induction is involved in the radiosensitization of talazoparib but is not the main action.

### 3.3. Talazoparib Enhanced Radiation-Induced Senescence in A549 Cells on Day 3

**Figure 3:**
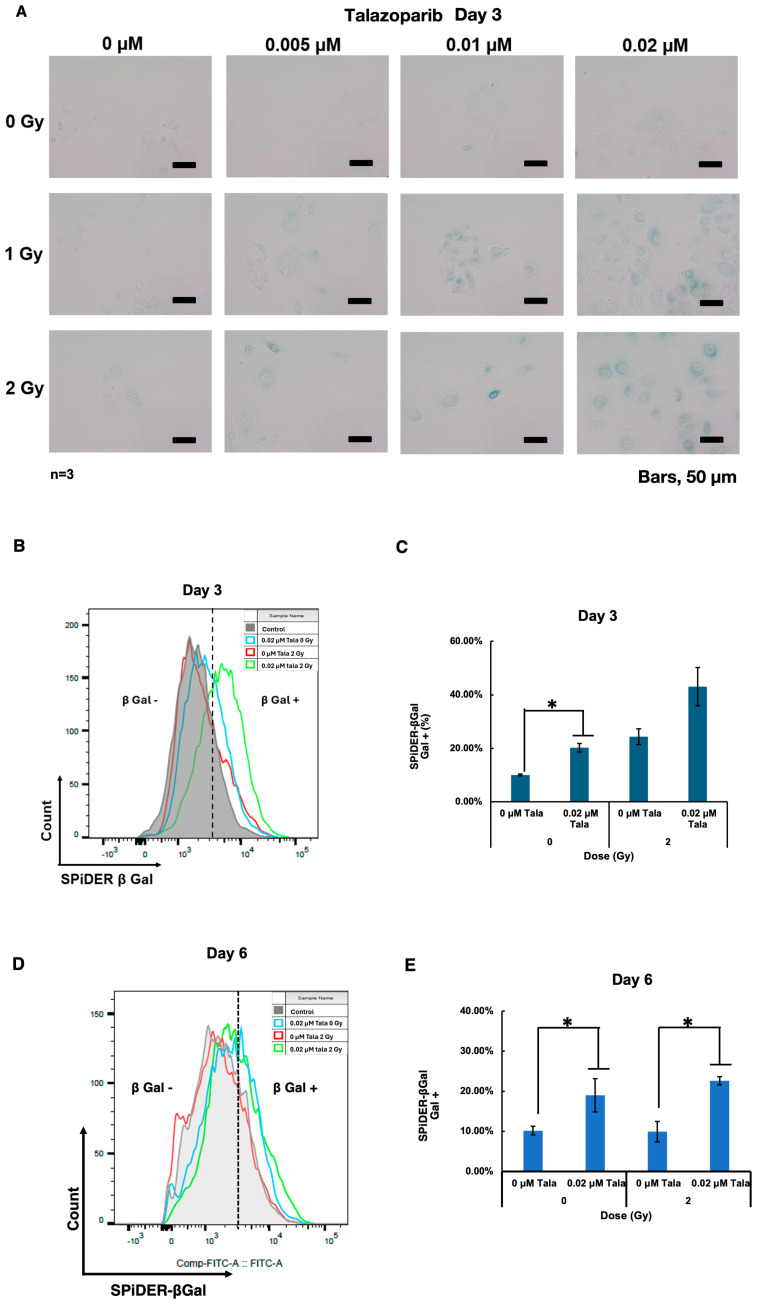
Talazoparib enhanced γ-irradiation-induced cellular senescence in A549 cells on day 3. Cellular senescence was analyzed by SA-β-galactosidase activity on day 3. SA-β-galactosidase activity was evaluated using X-gal as the substrate. A green color indicates that cells are senescence-positive. (**A**) Images were captured using a microscope on day 3. The scale bar represents 50 µm. (**B**–**E**) A SPiDER-β-gal assay was conducted on days 3 (**B**) and 6 (**D**). The typical results of flow cytometry profiles are shown. The percentage of SPiDER-β-gal-positive populations are shown on days 3 (**C**) and 6 (**E**). Biological replicates of *n* = 3, Mean ± SE. * *p* < 0.05.

Because it is known that cellular senescence induction causes radiosensitization in particular cases [13], we analyzed cellular senescence after the combined treatment with talazoparib and γ-irradiation using SA-β-galactosidase, which is a marker for cellular senescence. It was found that on day 3, induction of cellular senescence was not clearly observed with the talazoparib treatment alone, but it was slightly observed that γ-irradiation at 2 Gy and talazoparib concentration-dependently enhanced senescence induction (Figure 3A).

To quantify the level of senescence induction, we performed a further SPiDER-β-gal assay and analyzed the results with flow cytometry. The results showed that either talazoparib treatment or γ-irradiation alone induced cellular senescence, and γ-irradiation in combination with talazoparib enhanced cellular senescence on day 3 (Figure 3D). A similar enhancement by talazoparib on cellular senescence without γ-irradiation or after 2 Gy of irradiation was also observed on day 6 (Figure 3E).

### 3.4. Talazoparib Enhances the Decrease in Mitochondrial Membrane Potential After γ-Irradiation in A549 Cells

To understand the senescence enhancement mechanism of talazoparib after γ-irradiation, we examined the change in mitochondrial membrane potential using fluorescent dye JC-1 on chamber slides. The ratio of red (585 nm) to green (530 nm) fluorescence represents the changes in mitochondrial membrane potential. Normal mitochondria with mitochondrial membrane potential show red fluorescence at the polymerized state of this dye. When the mitochondrial potential is decreased, the cell shows green fluorescence because JC-1 localizes in the cytoplasm in the monomer state.

**Figure 4:**
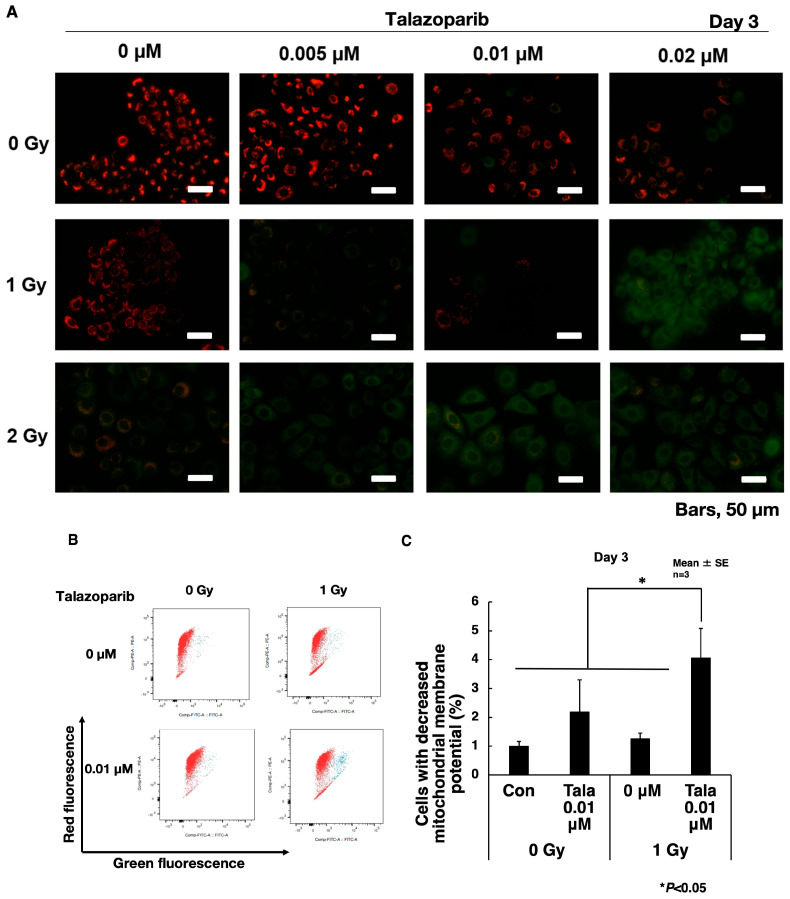
Reduced mitochondrial membrane potential on day 3 after a combined treatment of γ-irradiation and talazoparib in A549 cells. (**A**) Cells grown on slide glasses were treated with talazoparib and γ-irradiation and images were taken by fluorescence microscope on day 3 after treatment. Cells were treated with JC-1 dye for the detection of mitochondrial membrane potential. Red color shows normal mitochondrial membrane potential and green color shows reduced mitochondrial membrane potential. Scale bar: 50 µm. (**B**) Mitochondrial membrane potential was analyzed by flow cytometry of JC-1-stained cells after treatment with talazoparib on day 3 in A549 cells. Representative flow cytometry profiles of mitochondrial membrane potential. The red and green fluorescence shows JC-1 dye of the polymerized state in mitochondria and monomer state localized in the cytoplasm, respectively. (**C**) Quantified percentage of cells with reduced mitochondrial membrane potential by flow cytometry on day 3 after combined treatment with talazoparib and γ-irradiation. Biological replicates of *n* = 3. Mean + SE. * *p* < 0.05.

As shown in Figure 4A, after γ-irradiation, mitochondrial membrane potential was dose-dependently reduced in A549 cells on day 3. The combination of talazoparib in the range of 0.005–0.02 μM augmented the decrease in mitochondrial membrane potential concentration-dependently. To further quantify the changes in mitochondrial membrane potential, we used flow cytometry and measured the percentages of cells, which show a decrease in mitochondrial membrane potential (Figure 4B,C). The combination of talazoparib treatment with γ-irradiation at 1 Gy caused a reduction in mitochondrial membrane potential (*p* < 0.05).

### 3.5. p21 Knockdown Attenuated Cellular Senescence and Decrease in Mitochondrial Membrane Potential 

**Figure 5:**
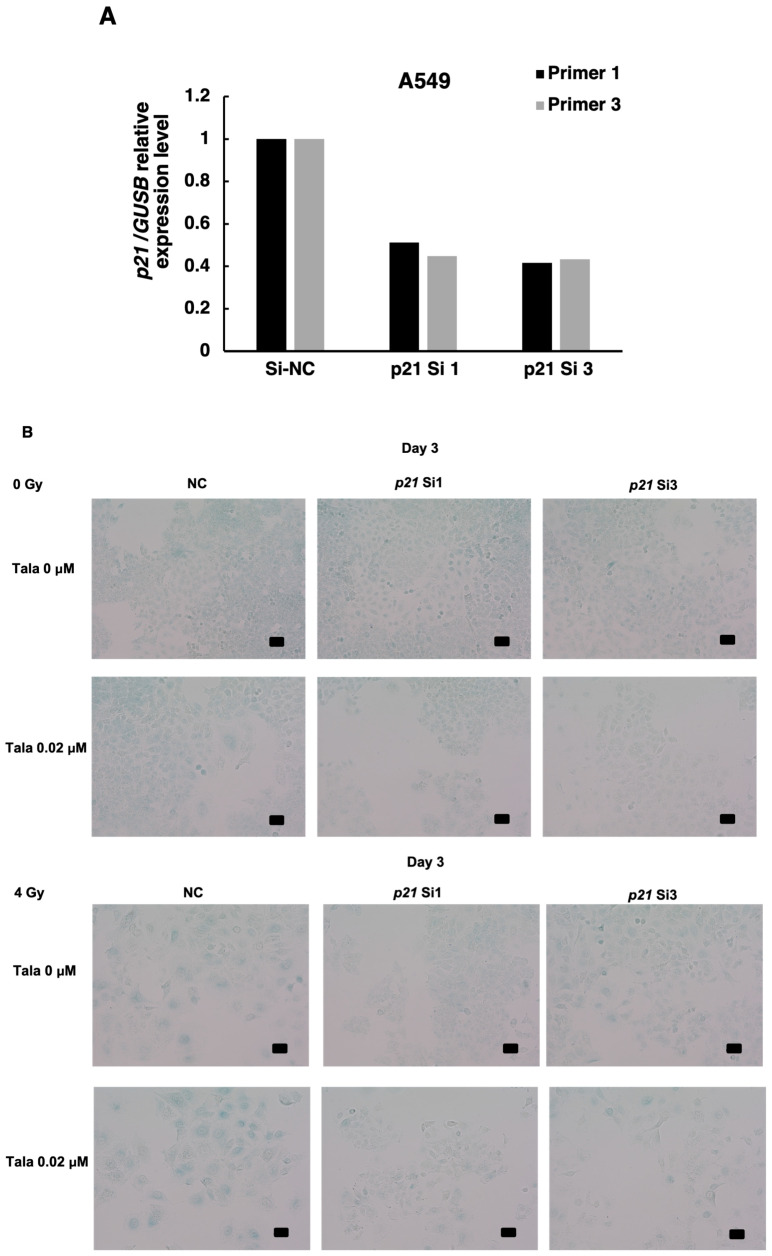

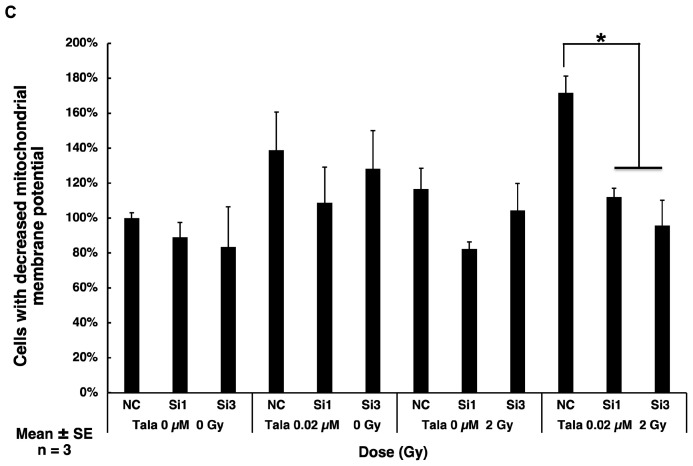
Cellular senescence induced by combined γ-irradiation and talazoparib treatment was reduced with *p21* knocked down in A549 cells. (**A**) Knocked down level of *p21* using two siRNAs, si1 and si3, analyzed by RT-PCR. *n* = 1. (**B**) Analysis of senescence after *p21* knockdown in mock-irradiated and irradiated A549 cells on day 3 by SA-β-galactosidase activity. Scale bar: 50 µm. (**C**) Mitochondrial membrane potential was analyzed by flow cytometry in JC-1-stained cells on day 2 in A549 cells. Representative flow cytometry profiles of mitochondrial membrane potential were shown in Supplemental Figure S2. A quantified percentage of cells with reduced mitochondrial membrane potential by flow cytometry on day 2. Biological replicates of *n* = 3. Mean + SE. * *p* < 0.05.

It is reported that cellular senescence was induced through a *p53*-dependent pathway with downstream factors of *p21* or *p16* in some cancers. In A549 cells, it is known that the *p16* gene has deletion mutation and is inactivated [19]. Therefore, it is speculated that *p21* could be responsible for the induction of cellular senescence. We thus investigated whether *p21* is involved in the induction of cellular senescence after γ-irradiation in combination with talazoparib. We then knocked down *p21* in A549 cells with two different siRNAs; the knockdown efficiency of *p21* was 60%. As shown in Figure 5A,B, when we analyzed cellular senescence after the combined treatment with talazoparib and γ-irradiation at 4 Gy using SA-β-galactosidase, we observed that the frequencies that were SA-β-galactosidase-positive were decreased by the transfection of *p21* siRNA (si1 and si3)-treated cells (Figure 5B). It was also observed that in *p21*-knocked down samples, the larger and flatter senescent cells were decreased.

We further examined whether *p21* knockdown affects mitochondrial membrane potential following γ-irradiation combined with talazoparib treatment. As shown in Figure 5C, on day 2, the decrease in the mitochondrial membrane potential was observed after treatment with talazoparib alone and more so in combination with γ-irradiation, whereas *p21* knockdown caused a significant decrease in mitochondrial membrane potential (Supplemental Figure S2).

## 4. Discussion

In this study we compared the radiosensitization effects of various types of clinically used PARPis in lung cancer A549 cells and found that talazoparib showed radiosensitization effects at the lowest concentration. We further investigated how talazoparib caused radiosensitization and showed that talazoparib treatment in combination with γ-irradiation enhanced cellular senescence induction in lung cancer cells, involving a decrease in mitochondrial membrane potential and p21 function. We observed that apoptosis is only slightly increased by the combination of talazoparib. It is previously reported that PARP inhibitors commonly delay DNA double strand repair, which causes S/G2M-phase arrest and apoptosis induction [1,5]. This could be the part of the effect of talazoparib, but we found that S/G2M-phase arrest was not increased in combination with talazoparib. Therefore, we speculated that cellular senescence may be involved in the radiosensitization caused by talazoparib.

We used lung adenocarcinoma A549 as an experimental model. The merit of the use of A549 cells is that we previously performed a comprehensive analysis of the radiosensitization gene in A549 cells and therefore know the pathways affecting their radiation sensitivity. A549 retains a p53-p21 signal transduction pathway but experiences deletion of the *p16* gene. Therefore, for the cancer cells retaining a p53-p21 signal transduction pathway, radiosensitization could be caused by talazoparib through inducing cellular senescence. It may be worth analyzing the effect of the presence of the *p16* gene on the radiosensitization effect of talazoparib in other cancer cells.

The application of PARP inhibitors as radiosensitizers has been widely investigated, with consistent evidence showing enhanced DNA damage and reduced repair capacity in tumor cells [1,6,20,21]. In lung adenocarcinoma, where intrinsic radioresistance remains a major obstacle to treatment success, PARP inhibition offers a rational approach to amplify the cytotoxic effects of radiotherapy [22,23]. Studies in both preclinical and clinical settings, including combinations of PARP inhibitors with platinum-based therapies and radiation, demonstrate promising outcomes [24,25,26,27]. Nevertheless, the variability in tumor response highlights the complexity of integrating these strategies into clinical practice [28,29,30,31]. One of the central challenges is therapy-induced senescence. While senescence initially suppresses proliferation, research indicates that tumor cells, including those in lung adenocarcinoma, may escape this arrest and regain proliferative capacity [8,32,33,34]. This phenomenon has been observed in experimental models where senescent cancer cells re-enter the cell cycle and contribute to recurrence [14,35,36]. Such findings emphasize the need to better understand the molecular switches that govern senescence stability, particularly in the context of radiotherapy and PARP inhibition.

The interplay between DNA damage and metabolism also requires attention. Disruption of the mitochondrial membrane potential following PARP inhibition connects nuclear DNA repair to metabolic collapse [16]. In lung adenocarcinoma, metabolic flexibility allows survival under stress, but targeted disruption of mitochondrial function may enhance radiosensitivity [37,38]. Similarly, the regulation of p21 is crucial: while p21 induction enforces growth arrest and cellular senescence through p53 signaling, its dysregulation can allow tumor cells to escape senescence or evade apoptosis [39]. Understanding this balance in lung adenocarcinoma could help identify biomarkers of treatment response. Beyond PARP1/2, emerging data highlights roles for less-studied PARP family members. For example, PARP16 has been implicated in endoplasmic reticulum stress and the unfolded protein response [40,41]. Although its role in lung cancer radiosensitization has not been explored, such pathways may contribute to adaptive resistance and represent new therapeutic targets.

It is an open question why talazoparib enhanced radiation-induced senescence while olaparib did not. Both talazoparib and olaparib inhibit PARP catalytic activity, and they exhibit significant differences in PARP-DNA trapping and pharmacological properties. Talazoparib demonstrates markedly stronger PARP-trapping activity compared to olaparib in cellular assays, which is believed to enhance cytotoxicity by stabilizing PARP–DNA complexes. In contrast, olaparib is a weaker trapper despite possessing comparable low-nanomolar catalytic inhibition. The structural differences between talazoparib’s compact fused heterocycle and olaparib’s *N*-acylpiperazine/phthalazinone scaffold likely influence their binding geometry and dissociation kinetics, thereby contributing to differential trapping, although the precise atomistic basis remains under investigation. These mechanistic and pharmacokinetic differences—talazoparib’s longer half-life and greater hematologic toxicity versus olaparib’s shorter half-life and CYP3A metabolism [42,43,44]—may be relevant to the potential differences in enhancing radiation-induced senescence.

PARP inhibitors for radiosensitization, including for lung and breast cancers and head and neck cancers, have been studied and reported [28,29,45,46,47]. While these works show that PARP inhibitors enhance radiosensitivity in lung cancer, our study provides a novel mechanistic insight by linking talazoparib to mitochondrial membrane potential loss and p21-associated signaling in irradiated A549 cells. These pathways have not been systematically explored in prior reports.

In this study, we observed a reduction in the mitochondrial membrane potential following talazoparib treatment, indicating a potential direct or indirect mitochondrial response to PARP inhibition. However, we did not measure the levels of reactive oxygen species (ROS), ATP, or NAD^+^, and thus cannot conclusively attribute the observed effects to oxidative stress, energy depletion, or NAD^+^-related pathways. Previous studies have demonstrated that PARP inhibitors can prevent mitochondrial dysfunction through blocking NAD^+^ depletion and altering energy metabolism, whereas *p21* has been implicated in mitochondrial regulation. Olaparib exposure was reported to induce mitochondrial oxidative stress by elevating mitochondrial ROS levels and diminishing glutathione peroxidase activity. Additionally, olaparib induced mitochondrial fission by decreasing the average length of mitochondria in ovarian cancer cells [48]. It should be further explored whether talazoparib-induced radiosensitization caused mitochondrial changes mediated through metabolic or p21-dependent mechanisms for senescence induction and whether it is common to cancer cells and normal cells.

Due to limitations in scope, in vivo validation with mouse models was not conducted at this stage. Nevertheless, we are actively expanding our analyses to encompass additional NSCLC models with diverse genetic backgrounds, including p53-mutant lines, and we intend to assess the findings in xenograft models in future investigations.

Clinical translation remains both promising and challenging. Trials investigating olaparib and talazoparib in combination with radiotherapy have reported encouraging results, but dose-limiting toxicities and variable responses remain hurdles [5,6,27,30]. Broader clinical studies in thoracic oncology emphasize the need for precision approaches that account for tumor genetics, repair capacity, and microenvironmental influences [49,50,51,52,53,54,55].

In summary, while PARP inhibition is a powerful radiosensitization strategy in lung adenocarcinoma, its success depends on understanding the interconnected roles of senescence, mitochondrial dysfunction, p21 signaling, and underexplored family members such as PARP16. Integrating these insights with clinical data and novel combination approaches will be critical to achieving durable therapeutic benefits.

## 5. Conclusions

Taken together, we showed the potential use of PARP inhibitor talazoparib for radiosensitization, which acts by inducing cellular senescence involving *p21* function in lung cancer treatment. Understanding these mechanisms of action and pathways could be used to selectively induce cell death by radiosensitization, offering a targeted approach to cancer therapy. This approach holds promise for developing more effective and precise cancer treatment strategies.

## Figures and Tables

**Table 1:** Radiation doses in all assays.

Assay	Dose (Gy)				
Clonogenic survival assay	0	2	4	6	7
Cell cycle analysis	0	2	-	-	-
Senescence assay	0	1	2	-	-
Mitochondrial membrane potential	0	1	2	-	-

**Table 2:** Enhancement ratio (ER) of PARP inhibitors at 10% survival and PE (plating efficiency).

PARP Inhibitors	ER10	PE
Talazoparib (0.005 µM)	1.5	0.6
Olaparib (1.3 µM)	1.8	1.1
Rucaparib (2 µM)	2.8	0.5
ABT888 (3 µM)	1.4	0.8
Niraparib (0.4 µM)	1.4	0.9

## Data Availability

The original contributions presented in this study are included in the article/Supplementary Material. Further inquiries can be directed to the corresponding author.

## Supplementary Materials

The following supporting information can be downloaded at: https://www.mdpi.com/article/10.3390/cimb47110908/s1.

## Abbreviations

The following abbreviations are used in this manuscript:

PARP	Poly(ADP-ribose) polymerase
JC-1	5,5, 6,6’-tetrachloro-1,1’, 3,3’-tetraethyl benzimidalyl carbocyanine iodide
S.E.	Standard error
ROS	Reactive oxygen species
