# Supplementary material for: Radiosensitization Effect of PARP Inhibitor Talazoparib Involves Decreasing Mitochondrial Membrane Potential and Induction of Cellular Senescence
Source: Curr Issues Mol Biol. 2025 Nov 1;47(11):908. doi: 10.3390/cimb47110908 (PMC12651182; doi:10.3390/cimb47110908)
Supplement: Supplementary file 1 [file cimb-47-00908-s001.zip › cimb-3921780-supplementary.pdf]

current issues in molecular biology

MDPI

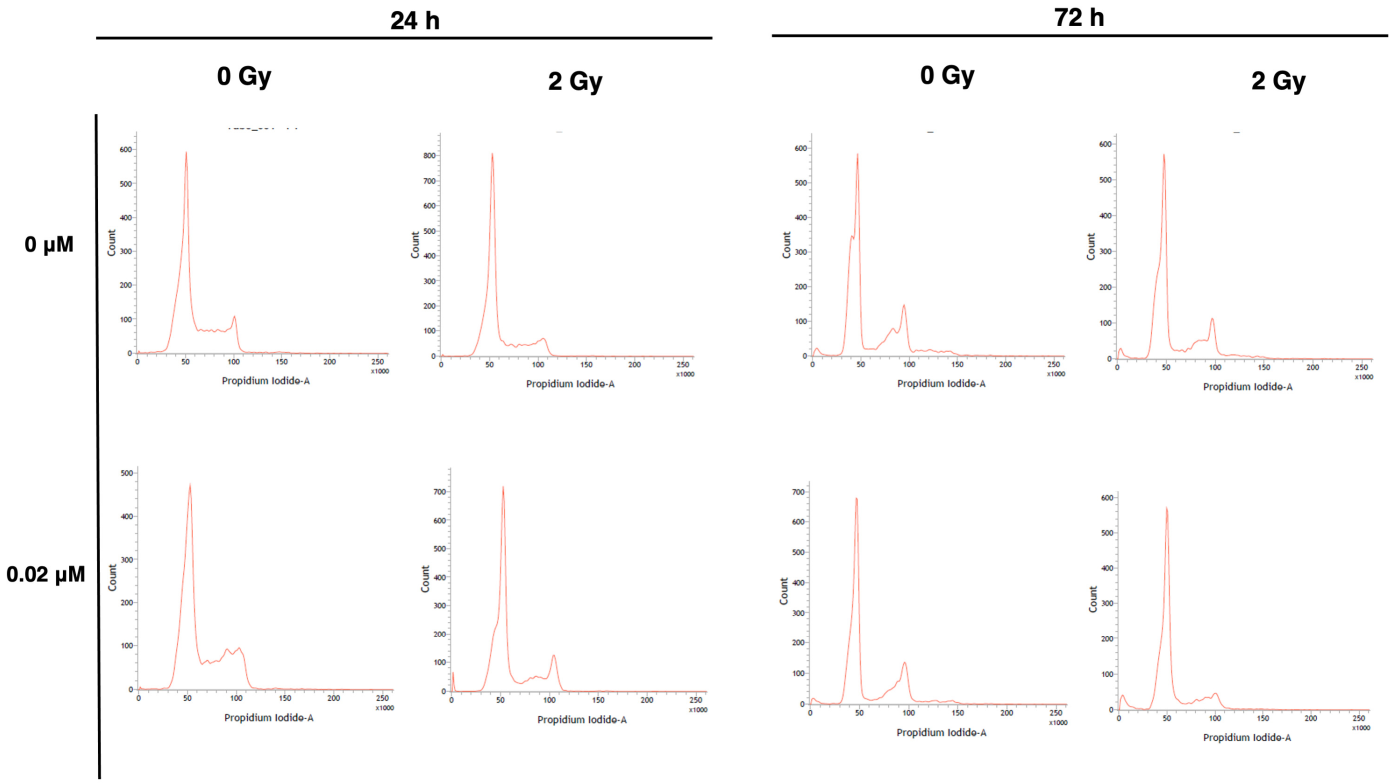

Figure S1: Cell cycle analysis after combined treatment with talazoparib & γ-irradiation at 24 h & 72 h in A549 cells;

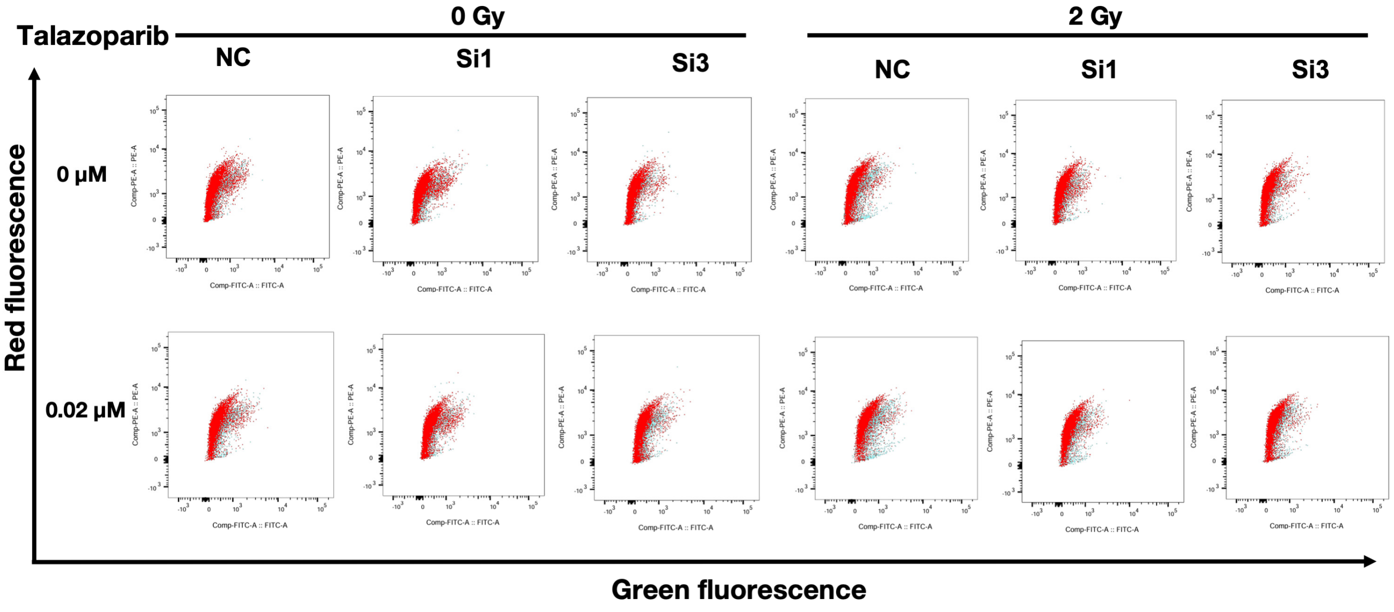

Figure S2: Analysis of mitochondrial membrane potential in A549 cells after knocked down of *p21* and combined treatment of talazoparib & γ-irradiation at day 2.
